# Evaluating the safety profile of calcineurin inhibitors: cancer risk in patients with systemic lupus erythematosus from the LUNA registry—a historical cohort study

**DOI:** 10.1186/s13075-024-03285-x

**Published:** 2024-02-12

**Authors:** Kunihiro Ichinose, Shuntaro Sato, Takashi Igawa, Momoko Okamoto, Ayuko Takatani, Yushiro Endo, Sosuke Tsuji, Toshimasa Shimizu, Remi Sumiyoshi, Tomohiro Koga, Shin-ya Kawashiri, Naoki Iwamoto, Mami Tamai, Hideki Nakamura, Tomoki Origuchi, Nobuyuki Yajima, Ken-Ei Sada, Yoshia Miyawaki, Ryusuke Yoshimi, Yasuhiro Shimojima, Shigeru Ohno, Hiroshi Kajiyama, Shuzo Sato, Michio Fujiwara, Atsushi Kawakami

**Affiliations:** 1 Department of Immunology and Rheumatology, Advanced Preventive Medical Sciences, Graduate School of Biomedical Sciences, Nagasaki University, Nagasaki, Japan; 2 Department of Rheumatology, Shimane University Faculty of Medicine, 89-1 Enya-Cho, Izumo, 693-8501 Japan; 3 Clinical Research Center, Nagasaki University Hospital, Nagasaki, Japan; 4 Department of Medicine, Division of Hematology and Rheumatology, Nihon University School of Medicine, Tokyo, Japan; 5 Department of Rehabilitation Sciences, Nagasaki University Graduate School of Biomedical Sciences, Nagasaki, Japan; 6 Department of Internal Medicine, Division of Rheumatology, Showa University School of Medicine, Shinagawa-Ku, Tokyo, Japan; 7 Department of Clinical Epidemiology, Kochi Medical School, Kochi University, Nankoku, Japan; 8 Department of Nephrology, Rheumatology, Endocrinology and Metabolism, Okayama University Graduate School of Medicine Dentistry and Pharmaceutical Sciences, Okayama, Japan; 9 Department of Stem Cell and Immune Regulation, Yokohama City University Graduate School of Medicine, Yokohama, Japan; 10 Department of Medicine (Neurology and Rheumatology), Shinshu University School of Medicine, Matsumoto, Japan; 11 Center for Rheumatic Diseases, Yokohama City University Medical Center, Yokohama, Japan; 12 Department of Rheumatology and Applied Immunology Faculty of Medicine, Saitama Medical University, Saitama, Japan; 13 Department of Rheumatology, Fukushima Medical University School of Medicine, Fukushima, Japan; 14 Department of Rheumatology, Yokohama Rosai Hospital, Yokohama, Japan

**Keywords:** Systemic lupus erythematosus, Calcineurin inhibitors, Cancer, Propensity score, IPW

## Abstract

**Background:**

Previous studies have shown conflicting evidence regarding the incidence of cancer in patients with systemic lupus erythematosus (SLE) compared with that in healthy individuals. Calcineurin inhibitors (CNIs) such as cyclosporine and tacrolimus have been widely used to treat SLE; however, their effects on cancer risk remain unclear. We aimed to investigate the incidence of cancer in patients with SLE and determine the potential association between CNI use and cancer risk.

**Methods:**

The standardized incidence ratio (SIR) of cancer among patients with lupus in the Lupus Registry of Nationwide Institutions (LUNA) was calculated based on the age-standardized incidence rate of cancer reported by Japan’s Ministry of Health, Labour and Welfare. We also examined the association between CNI exposure and cancer risk, while considering potential confounding factors. The analysis accounted for confounding variables such as age, sex, smoking history, maximum glucocorticoid dose, treatment history with cyclophosphamide, ongoing hydroxychloroquine, Systemic Lupus International Collaboration Clinics/American College of Rheumatology Damage Index (SDI) value (excluding cancer occurrence), comorbidity of diabetes mellitus, and smoking history.

**Results:**

The study included 704 patients with SLE (625 females; 88.8%) with a median age of 44 years [interquartile range (IQR) = 34–55] years. The median past maximum glucocorticoid dose was 40 mg/day [IQR = 30–60 mg/day], and the SDI at registration was 1 [IQR = 0–2]. Among the patients, 246 (35.1%) had smoking histories, and 38 (5.4%) experienced cancer complications. Gynecological malignancies accounted for 63.2% of all cancers. The SIR of cancer in the LUNA cohort was 1.08 (95% confidence interval [CI] = 0.74–1.43). No statistically significant risks of cancer were found in relation to CNI treatment history; the odds ratio using multiple logistic regression was 1.12 (95% CI = 0.42–3.00), the risk ratio using standardization was 1.18 (95% CI = 0.47–2.16), and the risk ratio using inverse probability weighting was 1.8 (95% CI = 0.41–4.66).

**Conclusions:**

The incidence of cancer in patients with SLE in the LUNA cohort did not significantly differ from that in the general population. These findings suggest that CNI treatment in this cohort did not pose a risk factor for cancer development.

**Supplementary Information:**

The online version contains supplementary material available at 10.1186/s13075-024-03285-x.

## Background

Systemic lupus erythematosus (SLE) and other autoimmune diseases are associated with an increased risk of certain types of cancers, although the results are inconsistent [1, 2]. Several extensive SLE cohort studies and meta-analyses have suggested that the rate at which patients with SLE are diagnosed with cancer is 14–76% higher than that in the general population [3]. In particular, patients with SLE may be at an increased risk for other cancers, such as lung, liver, cervical, and hematologic cancers, including non-Hodgkin lymphoma, Hodgkin lymphoma, and leukemia [4–7]. Many studies on putative risk factors for malignancies in this population have been conducted in response to the distinct cancer risk profiles of patients with SLE. Immunosuppressive medications, SLE disease activity, immunological abnormalities, viral and hormone exposure, autoantibodies, genetics, and many other variables increase the risk of malignancies in these patients [3].

Treatment with immunosuppressive or cytotoxic agents, such as hydroxychloroquine (HCQ), azathioprine, cyclophosphamide (CYC), methotrexate (MTX), and mycophenolate mofetil (MMF), is often combined with systemic glucocorticoids in patients with refractory symptoms or major organ involvement [8]. CYC is known to increase the risk of cancer associated with SLE, while HCQ decreases it [9]. Calcineurin inhibitors (CNIs), such as cyclosporine (CsA) and tacrolimus (TAC), have been postulated as potential treatment strategies for SLE and lupus nephritis (LN) because of the ability of these drugs to reduce T cell activation and promote immunosuppression [10]. In contrast, the chronic use of CsA and TAC has been reported to increase cancer incidence in patients who have received solid organ transplants [11]. Previous studies have indicated that CNIs can affect DNA repair mechanisms and promote angiogenesis and the invasion of non-metastatic cells in vitro [12, 13]. Despite advances in our knowledge of the processes that affect cancer risk in patients with SLE, the relationship between CNIs and cancer onset remains unclear.

In the present study, we calculated the standardized incidence ratio (SIR) of cancer among patients with lupus in the Lupus Registry of Nationwide Institutions (LUNA) based on the age-standardized incidence rate of cancer reported by Japan’s Ministry of Health, Labour and Welfare. We also investigated whether CNIs are associated with an increased risk of developing cancer.

## Patients and methods

This historical cohort study used a cohort from the LUNA, in which 10 Japanese institutions participated. This registry was established in 2016 to examine the associations among clinical presentation, serological testing, socioeconomic background, and outcomes in patients with SLE. The LUNA registry contains data on patients aged 20 years and older diagnosed with SLE using the 1997 American College of Rheumatology (ACR) criteria [14]. Approximately 900 cases (1.5%) of patients with SLE in Japan have been reported in LUNA.

All patients participating in the LUNA registry provided written informed consent, and the opt-out strategy was chosen for individual studies. Patients who refused to provide informed consent were excluded.

This study was performed in accordance with the Declaration of Helsinki and approved by the Investigation and Ethics Committee of Nagasaki University Hospital (approval nos. 18061802 and 20021020). Informed consent was obtained from all participants before enrollment in the study, and patient information was anonymized and de-identified before analysis.

### Data collection

This study used information from electronic medical records from February 2016 to September 2019. The data included laboratory tests, medications, activity scores, and comorbidities. We used identical methods to collect data on cancer development from the past to the time of the last observation using self-administered questionnaires completed by registered patients. Data were collected annually until the patient died, was discharged from the hospital, or consented to withdrawal from the registry. Demographic data included age at enrollment to the LUNA registry; age at diagnosis of SLE; sex; SLE Disease Activity Index 2000 (SLEDAI-2 K) [15]; The Systemic Lupus International Collaborating Clinics/ACR Damage Index (SDI) [16] (excluding the occurrence of cancer); comorbidities of LN, diabetes mellitus (DM), hypertension (HT), and hyperlipidemia (HL); habitual drinking; smoking history; cervical cancer vaccination history; maximum glucocorticoid dose (mg/day); and the use of immunosuppressant treatment.

### Exposure

Exposure was defined as continuous CNI (CsA and TAC) use from the time of SLE diagnosis to the time of study registration. Patients with cancer that developed before CNI treatment or those whose onset date was unknown were excluded.

### Study objective and outcome measures

The objective was to assess the safety of CNIs in patients with SLE with the primary outcome being the incidence of malignancies in these patients. This was evaluated by tracking the occurrence of various cancers (excluding cervical dysplasia) from the initiation of CNI therapy until the time of LUNA registration.

### Potential confounders

The following nine variables were identified as potential confounders based on findings from a previous study [1] and the clinical standpoint of the rheumatologist: (1) age at enrollment in the LUNA registry, (2) age at the time of diagnosis, (3) sex, (4) maximum glucocorticoid dose, (5) CYC treatment history, (6) ongoing HCQ, (7) SDI value (excluding the occurrence of cancer), (8) DM comorbidity, and (9) smoking history. All potential confounders were assessed and documented at registration. Items (4), (5), (8), and (9) included events from SLE onset until the time of registration; items (1), (2), (4), and (7) were continuous variables; and the remaining items were binary variables. A design diagram is shown to clarify the key points of each item (Additional file 1: Figure S1).

### Statistical analyses

First, the background characteristics of the LUNA cohort with and without cancer were summarized and compared using Wilcoxon’s rank sum and Fisher’s exact tests. Second, we calculated the SIR of cancer among patients with SLE based on the age-standardized incidence rate of cancer in 2016 as reported by the Cancer Statistics, Cancer Information Service, National Cancer Center, Japan National Cancer Registry, and Ministry of Health, Labour and Welfare. Third, we evaluated whether the history of CNI treatment was associated with cancer risk. Relative risk was estimated using crude analysis, a multivariable logistic regression model, standardization using propensity scores (PSs), and inverse probability weighting (IPW) to account for confounders [17]. Multiple imputations were used because data were missing. There were 100 imputed datasets, and the estimates from each dataset were combined using Rubin’s rules. For standardization and IPW using PSs, the PSs were first calculated for the 100 imputed datasets. Adjusted estimates were then calculated for each dataset and finally combined [18]. The 95% confidence intervals (CIs) were derived using the bootstrap method with 1000 iterations. See the Additional file 2 for details of the analysis method.

All statistical analyses were performed using JMP® Pro16 (SAS Institute, Cary, NC, USA) and R Statistical Software (Foundation for Statistical Computing, Vienna, Austria). A *p*-value was considered statistically significant when it was less than 0.05 (two-tailed) or when the 95% confidence interval did not include the null value.

## Results

### Patient characteristics in the study

**Fig. 1:**
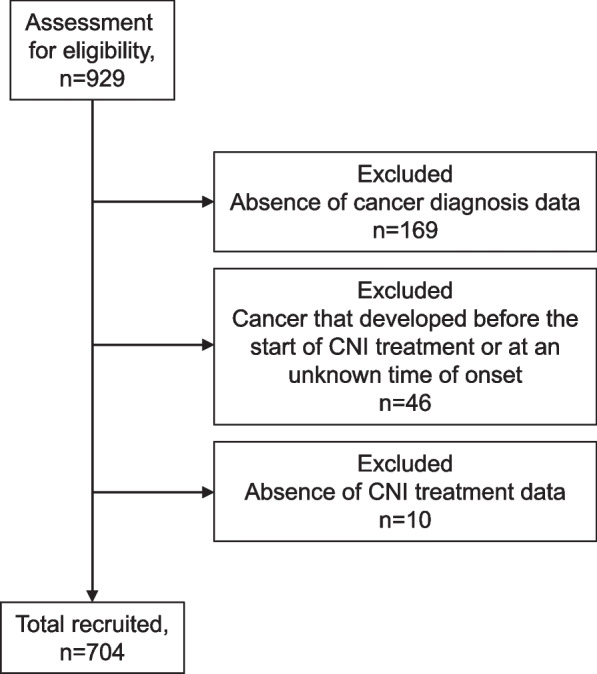
Patient enrollment flow diagram: 704 patients with SLE were enrolled. CNIs, calcineurin inhibitors

Of the 929 enrolled patients, 704 were followed up for the occurrence of cancer (Fig. 1). Thirty-eight cancer cases (5.4%), excluding cervical dysplasia, were identified within a median of 14 years from SLE onset to registration (Table 1). Patients with cancer were significantly older and had higher SDI scores (without cancer) at registration than those without cancer. In addition, a higher percentage of patients with cancer had DM and a history of smoking.

**Table 1:** Background characteristics of the patients in this study

Baseline variables	All patients (*n* = 704)		Cancer cases (*n* = 38)		Cancer-free cases (*n* = 666)		*p*-value
	Median	IQR	Median	IQR	Median	IQR	
Age at registration, years	44	34–55	50	42–65	44	34–55	< 0.001*
Age at SLE diagnosis, years	30	21–41	31	25–50	30	21–41	0.078
Sex (% female)	625 (88.8)		34 (89.5)		591 (88.7)		1.000
SELENA-SLEDAI at SLE diagnosis	14	(9–20)	13	(9–22)	14	(9–19)	0.980
SDI (without cancer) at registration	0	0–0	1	0–1	0	0–0	< 0.001*
Comorbidity of lupus nephritis	248 (36.0)		16 (42.1)		232 (35.7)		0.487
Comorbidity of diabetes mellitus	44 (7.0)		6 (16.7)		38 (6.4)		0.033*
Comorbidity of hypertension	231 (32.8)		13 (34.2)		218 (32.7)		0.860
Comorbidity of hyperlipidemia	174 (24.8)		11 (30.2)		161 (24.4)		0.408
Habitual drinking	67 (15.2)		7 (30.4)		60 (14.4)		0.065
Smoking history	246 (35.1)		25 (65.8)		221 (33.4)		< 0.001*
History of cervical cancer vaccination	35 (5.2)		0 (0.0)		35 (5.5)		0.250
Maximum glucocorticoid dose (mg/day)	40	30–60	50	30–60	40	30–60	0.157
IVCY	165 (23.5)		8 (21.6)		157 (23.6)		1.000
CNIs	91 (12.9)		6 (15.8)		85 (12.8)		0.617
MMF	44 (6.3)		3 (7.9)		41 (6.2)		0.725
MZR	106 (15.1)		4 (10.5)		102 (15.3)		0.639
MTX	25 (3.6)		1 (2.6)		24 (3.6)		1.000
AZP	112 (15.9)		6 (15.8)		106 (15.9)		1.000
HCQ	193 (27.5)		7 (18.4)		186 (28.0)		0.262

*p*-values were estimated using the nonparametric Wilcoxon’s rank-sum and Fisher’s exact tests

^*^p < 0.05. *IQR* interquartile range, *IVCY* intravenous cyclophosphamide, *CNIs *calcineurin inhibitors*, MMF* mycophenolate mofetil, *MZR* mizoribine, *MTX *methotrexate*, AZP* azathioprine, *HCQ* hydroxychloroquine, *SLE* systemic lupus erythematosus, *SELENA-SLEDAI* Safety of Estrogen in Lupus National Assessment—Systemic Lupus Erythematosus Disease Activity Index, *SDI* standardized incidence ratio

### Frequency and types of cancer

Thirty-eight patients with SLE (including 34 females) had cancers. There were 10 (26.3%) patients with cervical cancer, 6 (15.8%) with breast cancer, 6 (15.8%) with uterine cancer, and 2 (5.3%) with ovarian cancer. Among non-reproductive organ cancers, 3 (7.9%) were malignant lymphomas, 3 (7.9%) were gastric cancers, 2 (5.3%) were colon and rectal cancers, and 2 (5.3%) were kidney cancers.

### The SIR of cancer among patients with SLE

The SIR of cancer among patients with SLE was calculated based on the age-adjusted incidence of malignancies for each age group (Ministry of Health, Labour and Welfare, 2016) (Table 2). The SIRs of overall malignancies in total and in female patients with SLE compared with the general population were 1.08 (95% confidence interval [CI] = 0.74–1.43) and 1.18 (95% CI = 0.81–1.56), respectively. The incidence of malignancies in the LUNA cohort did not differ from that in the general population.

**Table 2:** Standardized incidence ratios (SIRs) and 95% confidence intervals (CIs) of cancers in patients with systemic lupus erythematosus (SLE), total and females

	No. of observations	Expected value	SIR	95% CI	*p*-value
Total	38	35.1	1.08	0.74–1.43	0.649
Female	34	28.7	1.18	0.81–1.56	0.321

### Crude risk difference and risk ratio of cancer development

Next, we examined the crude difference and risk ratio for developing cancer with and without CNI use (Table 3). Six patients (6.6%) in the CNI group and 32 patients (5.2%) in the non-CNI group developed cancer. The crude risk difference was 1.37 (95% CI =  − 4.02 to 6.77), and the risk ratio was 1.26 (95% CI = 0.54–2.94). The risk of developing cancer with CNI use in the LUNA cohort was not significantly different from that without CNI use.

**Table 3:** Crude risk difference and risk ratio of cancer development by calcineurin inhibitor use

Outcome	No (risk%)		Estimate (95% CI)	
	CNI use	No CNI use	Risk difference	Risk ratio
Cancer cases	6 (6.6)	32 (5.2)	1.37 (-4.02–6.77)	1.26 (0.54–2.94)

*CNIs* calcineurin inhibitors, *CI* confidence interval

### Adjusted odds/risk ratios and risk differences between CNI and non-CNI users

We then examined the odds/risk ratios and differences between CNI and non-CNI users after adjusting for potential confounders in the logistic regression analysis model, standardization, and IPW (Table 4). The odds ratio with the logistic model was 1.12 (95% CI = 0.42–3.00), the risk difference with standardization was 0.01 (95% CI =  − 0.04 to 0.06), the risk difference with IPW was 0.05 (95% CI =  − 0.04 to 0.24), the risk ratio with standardization was 1.18 (95% CI = 0.47–2.16), and the risk ratio with IPW was 1.8 (95% CI = 0.41–4.66). Even after adjusting for the confounders, the risk of developing cancer with CNIs did not differ significantly from that without CNIs in this study.

**Table 4:** Adjusted odds/risk ratios and risk differences between CNI users and non-CNI users after adjustments for the logistic regression analysis, propensity scoring, and IPW

Effect	Estimate	(95% CI)
Odds ratio with outcome model	1.12	(0.42–3.00)
Risk difference with standardization	0.01	(− 0.04 to 0.06)
Risk difference with IPW	0.05	(− 0.04 to 0.24)
Risk ratio with standardization	1.18	(0.47–2.16)
Risk ratio with IPW	1.8	(0.41–4.66)

*CNI* calcineurin inhibitor, *IPW* inverse probability weighting, *CI* confidence interval

## Discussion

Previous studies have shown conflicting evidence regarding the incidence of cancer in patients with SLE compared with that in healthy individuals. CNIs such as CsA and TAC have been widely used to treat SLE; however, their effects on cancer risk remain unclear. We aimed to investigate the incidence of cancer among patients with SLE in the LUNA cohort and the possible association between CNI treatment and cancer development. We found that the incidence of cancer in patients with SLE in the LUNA cohort did not significantly differ from that in the general population. Furthermore, our results suggest that CNI treatment in the LUNA cohort does not significantly increase the risk of cancer development. However, we recognize the importance of cautious interpretation and the need for further studies to confirm these findings in broader contexts.

Our first finding revealed that the incidence of cancer in patients with SLE in the LUNA cohort did not differ from that in the general population. There are conflicting views on whether SLE increases cancer risk. Some studies have suggested that patients with SLE may experience an elevated risk of certain types of cancer, such as lymphoma and cancer of the cervix [5]. However, other sources suggest that having SLE does not significantly increase the risk of common cancers such as breast, uterine, ovarian, pancreatic, colon, and brain cancers and that the overall increased cancer risk is minimal [1, 2, 19]. One study found that patients with SLE have a decreased risk of certain cancers, such as prostate and cutaneous melanoma [20]. In our study, there was a high incidence of cancer in the cervical region (26.3%), breast (15.8%), and uterus (15.8%) in the LUNA cohort. However, the risk of cancer in individual organs was not examined owing to the limited patient numbers and is a subject of future investigation. Overall, our study provides evidence that patients with SLE in the LUNA cohort do not develop cancer more often than the general population.

The second finding suggests that CNI treatment is unlikely to be a risk factor for cancer development. To date, no association has been demonstrated between oral CNIs and cancer development in patients with SLE. Previous studies have shown that the immunosuppressive effects of CNIs contribute to tumor development by decreasing cancer cell surveillance. Additionally, there is evidence of direct tumor induction by CNIs [21–23]. Skin cancer is associated with the use of CNIs in solid-organ transplantation [21]. This may result from the localized inhibition of DNA repair and apoptosis in the skin.

Alternatively, recent findings have shown with moderate certainty that topical CNIs do not increase the risk of cancer in patients with atopic dermatitis [24]. These findings support the safe use of topical CNIs for the optimal treatment of patients with atopic dermatitis. Furthermore, evidence indicates that medications including aspirin [25], nonsteroidal anti-inflammatory drugs [26], and glucocorticoids [27] may influence the risk of malignancy in SLE, although the data are mixed with some studies indicating a potential reduction in risk. Oral CNIs may modify the effects of these agents; however, our results may not indicate a direct effect of CNIs.

Our study makes a valuable contribution to the ongoing discussion regarding the safety of CNI treatment in patients with SLE. Our findings from the LUNA cohort indicate that patients with SLE do not exhibit a higher frequency of cancer development than the general population. Furthermore, our analysis suggests the lack of a strong association between CNI treatment and increased cancer risk in this specific cohort. This observation, while preliminary, may offer some reassurance to clinicians and patients about the use of CNIs in managing SLE and other autoimmune diseases. However, we emphasize the importance of further research to substantiate these findings and fully understand their clinical implications.

Our study has some limitations that should be considered. First, we analyzed only the LUNA cohort, which comprised patients from one geographical region, and our findings may not apply to other populations. Second, we could not evaluate the effects of the duration and dose of CNI treatment on the risk of cancer owing to the limited data. Therefore, the actual CNI exposure in patients may vary widely, and it may not be comparable between patients with cancer and cancer-free cases. Third, there is no established median period for the effects of CNI use on cancer development. Cancer that developed shortly after CNI initiation may not be linked to CNI. A sufficiently long follow-up period seems necessary to link cancer incidence and other outcomes in patients receiving CNI. Fourth, we could not investigate the potential effect of disease severity on the incidence of cancer in patients with SLE. Fifth, the LUNA cohort used HCQ less frequently (less than 30%), which may have led to a higher cancer incidence. Sixth, our results may have been altered by the many adjustment variables for cancer incidence outcomes. Finally, our study did not include other risk factors for cancer, such as other medications, food preferences, infectious diseases, obesity, and family history of cancer. These limitations may be overcome in the future as more patients are added through international collaborative studies involving SLE cohorts. Despite these limitations, our study has several strengths. First, we analyzed a large cohort of patients with SLE over a long follow-up period. Second, we used robust statistical methods to control confounding factors.

## Conclusions

In conclusion, our study provides preliminary evidence that the incidence of cancer in SLE patients within the LUNA cohort may not differ significantly from that in the general population. Furthermore, our data suggest that CNI treatment is not a substantial risk factor for cancer development in this cohort. While these observations could be informative for clinicians treating SLE patients with CNIs, we emphasize the need for further research to confirm these findings and fully understand their implications.

### Supplementary Information

**Additional file 1: Figure S1.** Design diagram to clarify the key points of each item.

**Additional file 2.** Supplement of statistical analyses.

## Data Availability

The dataset analyzed in this study is available from the corresponding author upon reasonable request.
