# Supplementary material for: Evaluating the safety profile of calcineurin inhibitors: cancer risk in patients with systemic lupus erythematosus from the LUNA registry—a historical cohort study
Source: Arthritis Res Ther. 2024 Feb 12;26:48. doi: 10.1186/s13075-024-03285-x (PMC10860233; doi:10.1186/s13075-024-03285-x)
Supplement: Supplementary file 1 — Additional file 1: Figure S1. Design diagram to clarify the key points of each item. [file 13075_2024_3285_MOESM1_ESM.pptx]

## Slide 1
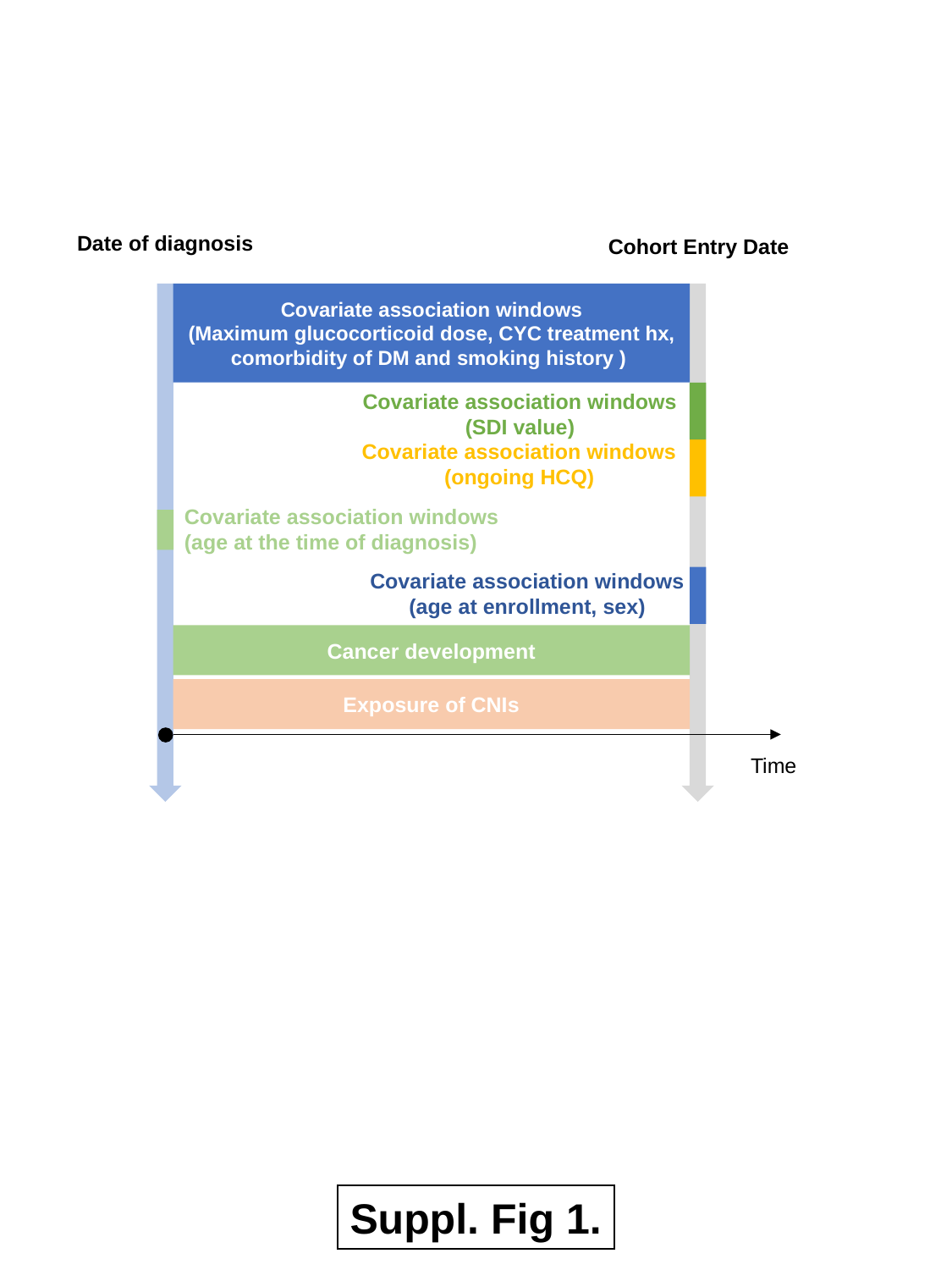

Date of diagnosis
Cohort Entry Date
Covariate association windows
(Maximum glucocorticoid dose, CYC treatment hx, comorbidity of DM and smoking history )
Covariate association windows
(SDI value)
Covariate association windows
(ongoing HCQ)
Covariate association windows
(age at the time of diagnosis)
Covariate association windows
(age at enrollment, sex)
Cancer development
Exposure of CNIs
Time
Suppl. Fig 1.
