# Supplementary material for: Evaluating the safety profile of calcineurin inhibitors: cancer risk in patients with systemic lupus erythematosus from the LUNA registry—a historical cohort study
Source: Arthritis Res Ther. 2024 Feb 12;26:48. doi: 10.1186/s13075-024-03285-x (PMC10860233; doi:10.1186/s13075-024-03285-x)
Supplement: Supplementary file 2 — Additional file 2. Supplement of statistical analyses. [file 13075_2024_3285_MOESM2_ESM.docx]

Supplement of statistical analyses

In this study, the following three analysis methods were used to evaluate the association between CNI treatment and cancer incidence, controlling for confounding factors.

1. Usual logistic regression model

2. Standardization with propensity score

3. Inverse probability weighting method using propensity scores

1 was the outcome model, and 2 and 3 the propensity score models.

The variables used in the model were as follows.

- Outcome variable: Cancer occurrence
- Exposure variable: CNI treatment
- Confounding factors: age at enrollment in the LUNA registry, age at the time of diagnosis, sex, maximum glucocorticoid dose, CYC treatment history, ongoing HCQ, SDI value (excluding the occurrence of cancer), comorbidity of DM, and smoking history.

In the outcome model, the outcome variable was included as the objective variable, and the exposure variable and confounding factors were included as explanatory variables. No interaction terms were included. In the propensity score model, the propensity score was estimated by a logistic regression model that included the exposure variable as the objective variable and confounding factors as explanatory variables. As in the outcome model, interaction terms were not included.

In particular, 2 and 3 estimated the average treatment effect, which is the estimand when the total population is hypothetically treated with and without CNI. 1's estimand was strictly different from 2 and 3's, but since we did not include an interaction term in the model, we can assume the average treatment effect. As noted in the “Causal Inference: What If” of Hernán and Robins. We present results from multiple methods to confirm the robustness of the estimated values.

Furthermore, missing values were present in this study. We accounted for missing values using the multiple imputation method and combined it with estimating effects using a model. Specifically, the following procedure was used with reference to Granger's paper. 1.

1. Create imputed data using the multiple imputation method

2. Estimate each imputed complete data using a model (logistic regression model, propensity score model)

3. Combine the estimates using Rubin's rule

1) Hernán MA, Robins JM. 2020. Causal Inference: What If. Boca Raton: Chapman & Hall/CRC.

2) Granger E, Sergeant JC, Lunt M. 2019. Avoiding pitfalls when combining multiple imputation and propensity scores. *Statistics in medicine* 38:5120–5132.
